# Feasibility of prehabilitation for patients awaiting total knee arthroplasty; a pilot study

**DOI:** 10.1016/j.jor.2024.07.019

**Published:** 2024-07-30

**Authors:** Louisa T.M.A. Mulder, Danielle D.P. Berghmans, Peter Z. Feczko, Rob A. de Bie, Antoine F. Lenssen

**Affiliations:** a Department of Epidemiology, CAPHRI School for Public Health and Primary Care, Maastricht University, P. Debyeplein 1, 6229 HA, Maastricht, the Netherlands; b Department of Physical Therapy, Maastricht University Medical Centre+, P. Debyelaan 25, 6229 HX, Maastricht, the Netherlands; c Department of Orthopedic Surgery, Maastricht University Medical Centre+, P. Debyelaan 25, 6229 HX, Maastricht, the Netherlands

**Keywords:** Preoperative exercise, Total knee arthroplasty, Feasibility studies

## Abstract

**Objective:**

To examine the feasibility of conducting a preoperative home-based prehabilitation program for total knee arthroplasty patients at risk for delayed in-hospital recovery, and to explore the pre- and postoperative impact of this program.

**Design:**

A retrospective cohort study with matched controls, enabling subgroup analyses.

**Setting:**

Home-based.

**Subjects:**

Patients awaiting primary unilateral total knee arthroplasty between 2019 and 2020, were compared with matched historic cases from 2016 to 2017. Matching criteria were scoring ≤17 points on the De Morton Mobility Index and >12.5 s on the timed-up-and-go test.

**Intervention:**

Supervised home-based prehabilitation program versus no prehabilitation.

**Outcomes:**

Feasibility, determined by recruitment rate, adherence, and safety of the program. Preoperative impact, assessed for the intervention group by differences in mean values for aerobic capacity, muscle strength and functional mobility between the first and last sessions. Postoperative impact was defined as the time needed to achieve in-hospital independence of physical function and was measured by the differences in mean values between the intervention and control groups.

**Results:**

Recruitment rate was 71 %; adherence and drop-out rates were 88 % and 12 % respectively. No adverse events were reported. Preoperatively, the intervention group showed significant improvements in aerobic capacity on the 2-min walking test (84.29 m–98.06 m; p = 0.007) and 2-min step test (40.35 steps to 52.95 steps; p = 0.014), muscle strength on the 30 s chair stand test (7.3 stands to 10.1 stands; p = 0.002), and functional mobility as seen in the timed-up-and-go-test (19.52 s–15.85 s; p = 0.031). Postoperatively, the intervention group achieved in-hospital independence of physical function earlier (mean rank 16.11) than the control group (mean rank 24.89; p=<0.01).

**Conclusions:**

It is feasible to conduct a prehabilitation program 4–6 weeks preoperatively, for high-risk patients awaiting total knee arthroplasty. Additionally, the program appears to have a positive impact on physical fitness both pre- and postoperatively.

## 1 Introduction

Most patients with long-lasting end-stage knee osteoarthritis (KOA) experience reduced physical activity, deconditioning and fatigue, which impacts negatively upon participation in the community.^1^, ^2^, ^3^ If surgery is ultimately needed, these problems are likely to have negatively affected the preoperative physical fitness of these patients. With 21 444 total knee arthroplasty (TKA) surgeries in the Netherlands in 2021, this potentially affects a major patient group.^4^

It has been reported that preoperative physical fitness status is a strong determinant of postoperative physical function in patients with lower extremity total joint replacements.^5^, ^6^, ^7^ To improve patients' preoperative physical fitness, structured exercise programs, supervised by physical therapists, also called ‘prehabilitation’, have been designed and tend to improve function before as well as immediately after surgery.^8^ Prehabilitation enhances the body's ability to withstand the stress of surgery and subsequently leads to improved postoperative outcomes.^9^, ^10^, ^11^ It also creates a better starting position for postoperative rehabilitation. It is especially in high-risk patients that prehabilitation has been demonstrated to be effective in reducing the risk of postoperative complications and in attaining faster recovery of physical function.^12^ This has been found, for example, in patients awaiting elective colorectal cancer surgery.^13^^,^^14^ Several systematic reviews have been conducted regarding prehabilitation for KOA patients awaiting TKA surgery, or regarding elective orthopedic surgery in general.^8^^,^^15^ However, these studies did not specifically pre-select high-risk patients. Very little research has been done on the feasibility of prehabilitation for high-risk patients awaiting elective TKA.^16^ Nevertheless, several studies and guidelines have reported that it is the high-risk patient group, mostly older patients with multisystem impairments and increased disabilities, who may benefit the most from a home-based prehabilitation program.^3^^,^^10^^,^^17^

Therefore, the aim of this study was to examine the feasibility of a home-based prehabilitation program, starting 4–6 weeks before TKA in high-risk patients. Feasibility was examined by assessing the recruitment rate (% of eligible patients participating), adherence (% completing the program), safety (% dropout rate) and occurrence of adverse events related to the program. Secondly, it was investigated whether these patients were able to improve their physical fitness during the prehabilitation program, making them better prepared for elective surgery, compared to high-risk patients without prehabilitation. This was assessed preoperatively by measuring objective changes in aerobic capacity, muscle strength and functional mobility in the intervention group. Postoperatively, this was assessed by assessing the differences in the time needed to achieve in-hospital independence of physical function, for both the intervention group and the control group.

## 2 Methods

### 2.1 Design

A retrospective, matched control cohort design was used to answer the research questions. The data collection procedures adhered to the principles outlined in the Declaration of Helsinki and received approval from the Ethical Review Board of *[blinded version]*. Considering the retrospective study design where data collection occurred as part of routine care, informed consent was not applicable.

### 2.2 Participants

Patients were eligible if they had been selected for elective primary TKA due to KOA between 2016 and 2020, and scored ≤17 points on the De Morton Mobility Index^18^ (DEMMI), as well as having a timed-up-and-go^19^ (TUG) score of >12.5 s at the preoperative assessment. Patients who were pre-scheduled to attend a rehabilitation clinic for further recovery post-surgery were excluded from the study. This exclusion was made because they might leave the hospital before achieving independence in physical function. The inclusion and exclusion criteria are listed in Table 1.

**Table 1:** Inclusion and exclusion criteria.

Inclusion criteria	Exclusion criteria
TKA between 2016 and 2020 Performed the preoperative assessment Reason: TKA because of KOA DEMMI ≤17 points TUG >12.5 s	Unicompartmental or revision surgeries Predetermined discharge location being a rehabilitation center or nursing home

TKA = total knee arthroplasty; KOA = knee osteoarthritis; DEMMI = De Morton Mobility Index; TUG = timed-up-and-go test.

### 2.3 Procedure

The feasibility and preoperative impact of the prehabilitation program were examined using a cohort of patients scheduled for elective surgery in 2019–2020, referred to below as the intervention group. As part of the planned procedure, all patients visited the physical therapy department of *[blinded version]*, for preoperative physical function assessment 4–6 weeks before surgery. This assessment was part of the usual care protocol. Patients scoring 17 points or less on the DEMMI^18^ and having a TUG^19^ score of more than 12.5 s were considered to be at high risk for delayed recovery.^10^ These patients received a prehabilitation program, which was supervised by trained physical therapists.^10^ The program consisted of 4–6 weeks of home-based training (two to three times a week, each session lasting 30–45 min). Training goals were improvement of physiological reserve capacity and physical functioning. Therefore, the training program comprised a blend of functional exercises focusing on enhancing strength, mobility, and cardiovascular health. Physical parameters were monitored weekly to optimize the intensity of the program (Table 2). During the training, intensity was based on the patients’ ability, derived from a Borg Rating of Perceived Exertion (RPE) score around 15, which reflects a heavy level of physical activity.^10^^,^^20^ This was monitored after every exercise. Patients continued this program until hospitalization. After surgery, the number of days needed to achieve in-hospital independence of physical function was measured by the modified Iowa Level of Assistance Scale (mILAS).^21^

**Table 2:** Monitoring variables of prehabilitation.

Variables	Measurements
Aerobic capacity	2MST,^22^ 2MWT,^23^ 30CST^24^
Muscle strength	30CST^24^
Functional mobility	TUG^19^
Exertion	Borg-RPE^20^

2MST = 2-min step test; 2MWT = 2-min walking test; 30CTS = 30-s chair stand test; TUG = timed up-and-go test; Borg-RPE = Borg Rating of Perceived Exertion scale.

The postoperative impact of the prehabilitation program was based on the number of days needed to achieve in-hospital independence of physical function. A comparable group was based out of a cohort of patients scheduled for elective TKA surgery in 2016–2017, referred to below as the control group. All patients in this 2016–2017 cohort had been informed about their upcoming hospital stay and the importance of maintaining their physical fitness. This was done during the preoperative physical function assessment 4–6 weeks before surgery, just as in the 2019–2020 cohort. At that time, however, no supervised prehabilitation program was provided.

### 2.4 Pathway changes

A retrospective group from 2016 to 2017 was chosen because we had just concluded an extensive perioperative program reconstruction at the start of 2016. The entire care pathway was cleaned up (including preoperative screenings, same-day admission, start of therapeutic intervention on surgery day, twice-daily therapeutic intervention for patients going home). As a consequence the care structure in 2019–2020 is comparable to that of 2016–2017. During 2018, preparations were made to establish a physical therapy network and train the physical therapists. This can be seen as a transition year, hence no patients were included. An implementation period followed. Subsequently, prehabilitation of patients awaiting TKA could be started in 2019.

### 2.5 Preoperative assessment

De Morton Mobility Index (DEMMI) measures the level of independence in basic motor activities among older patients.^18^ Comprising 15 items, each item is scored on a 2- or 3-point scale (0–1 or 0–1–2), resulting in a total score ranging from 0 to 19 points. Higher scores indicate greater independence in functioning.^25^ The DEMMI is reproducible, valid, and feasible for patients with KOA, demonstrating an interrater reliability with an ICC of 0.85 (95 % CI 0.71–0.93).^25^

The timed-up-and-go test (TUG) evaluated functional mobility by measuring the time taken to stand up from a chair, walk 3 m, turn, walk back, and sit down.^19^ It's a commonly used measure for physical function in patients with KOA and TKA,^26^ showing excellent test-retest reliability (ICC = 0.97).^27^

### 2.6 Assessment during prehabilitation

The 2-min step test (2MST) involved patients lifting their knees within a 2-min timeframe to determine aerobic capacity. A reference mark on the wall, placed exactly midway between the patella and the spina iliaca anterior superior, guided the knee lifts. Successful repetitions required reaching this mark.^22^ The test demonstrated excellent inter-rater reliability (ICC = 0.97).^22^

The 2-min walking test (2MWT) measured aerobic capacity by having patients walk as far as possible along a 20-m corridor in 2 min, with walking aids permitted.^23^ Test-retest reliability was reported to be excellent.^28^

The 30 s chair stand test (30CST) assessed functional lower extremity strength and aerobic capacity. Patients aimed to perform as many sit-to-stand transfers as possible within 30 s, without using arm support.^24^ The test-retest reliability was excellent (r = 0.89, 95%CI 0.79–0.93).^24^

The timed-up-and-go test (TUG) was described in chapter 2.5: Preoperative assessment.

The Borg Rating of Perceived Exertion (Borg-RPE) score was used to assess the level of exertion.^20^ Immediately after the exercises, patients were asked to indicate their feeling of exertion as a score between 6 (no exertion at all) and 20 (extremely hard), reflecting the intensity of the exercise. The Borg-RPE has excellent test-retest reliability (ICC = 0.89).^29^

### 2.7 Variable monitored during hospitalization

The modified Iowa Level of Assistance Scale (mILAS) which assessed the assistance needed for activities of daily living. Scores ranged from 0 (independent) to 6 (unable for medical or safety reasons), with lower scores indicating greater independence.^21^ Climbing stairs, excluded for some patients, was not required. The mILAS exhibited high reliability, with an inter-rater reliability of 0.96 (ICC) (95%CI 0.93–0.98),^30^ and is considered valid and responsive for determining post-total knee arthroplasty independence levels.^21^

### 2.8 Primary outcome

This was feasibility of the prehabilitation program. Although feasibility studies focus on different aspects,^31^ the current study focused on the practicality of the program. This was assessed by the recruitment rate (% of eligible patients participating), adherence (% completing the program) and safety (% dropout rate and occurrence of program-related adverse events during the sessions). The program was deemed feasible if recruitment rate was ≥70 %, adherence was ≥80 %, dropout rate was ≤20 % and no adverse events occurred.^32^, ^33^, ^34^

### 2.9 Secondary outcomes

This was the impact of the prehabilitation program. This was divided into preoperative and postoperative impact. Preoperative impact was described in the intervention group as the change in aerobic capacity, muscle strength and functional mobility between the first and last sessions. Postoperative impact was defined as a shorter time needed to achieve in-hospital independence of physical function, measured by the mILAS. This was measured in all patients. To facilitate comparison between the two cohorts, and to avoid confounding by low-risk patients from the 2016–2017 cohort, patients with similar DEMMI scores from both cohorts were matched as closely as possible, i.e. matching scores should not differ by more than 1 point. If there was more than one possible match, matching TUG scores were assessed. These should not differ by more than 1 s between the control and intervention groups.

### 2.10 Statistical analysis

The data analysis utilized SPSS version 28.0 (IBM, New York, USA). Continuous variables were summarized using median and interquartile range (IQR), while categorical variables were represented by frequencies and percentages. Normality assumptions were assessed via histograms and the Shapiro-Wilk test (P ≤ 0.05). Data completeness was verified, and missing values (>5 %) were imputed using multiple imputation, employing predictive mean matching for continuous variables.^35^ Five imputations were conducted.^36^ A sensitivity analysis was carried out to ensure that imputation did not significantly alter the results.

Preoperative impact. For assessing changes within the intervention group, the paired samples *t*-test was employed for normally distributed data, while the Wilcoxon Signed Rank Test was utilized for nonparametric data (P ≤ 0.05).

Postoperative impact. For determining variances between the intervention and control groups, the independent samples *t*-test was applied for normally distributed data. In cases of non-normally distributed data, the Mann-Whitney *U* test was employed (P ≤ 0.05).

## 3 Results

### 3.1 Feasibility

**Fig. 1:**
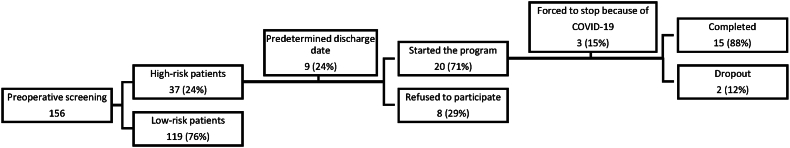
Flow-chart of patients in the intervention group; numbers reflect number of patients at each stage.

In the 2019–2020 cohort, a total of 156 patients were screened, 37 of whom (24 %) were classified as high-risk (TUG>12.5sec and DEMMI≤17). Eight (29 %) patients did not enter the program; reasons for their non-participation were not clear. Nine (24 %) patients had a predetermined discharge date and were therefore excluded. Twenty patients started the program, corresponding to a recruitment rate of 71 %. Three (15 %) were forced to stop because of the COVID-19 outbreak; these were excluded from the feasibility analysis. Fifteen patients completed the program until surgery, which corresponded to an adherence rate of 88 %. Two (12 %) patients stopped during the intervention. Reasons for stopping were increased pain and lack of motivation (Fig. 1). There were no reported adverse events throughout the program. The number of sessions followed varied between one and nine, with a median of 6 (3) sessions.

### 3.2 Patient characteristics

The intervention group (n = 20) was assembled based on patients from the 2019–2020 cohort, all patients who participated in the prehabilitation program being included. The control group (n = 20) consisted of matched patients from the 2016–2017 cohort. Despite intending to go home, six patients in the intervention group went to a rehabilitation center after surgery. In the control group 10 patients went to a rehabilitation center after surgery. Patient characteristics are shown in Table 3.

**Table 3:** Patient characteristics.

	Intervention group (n = 20)	Control group (n = 20)
Gender: male – female	4 (20) – 16 (80)	5 (25) – 15 (75)
Days to mILAS0^a^	2 (1)	4 (3)
Range of days to mILAS0	1–8	3–8
Age at screening	73.0 (10)	75.00 (14)
Height (m)	1.65 (0.1)	1.59 (0.1)
Weight (kg)	92.5 (19.2)	89.0 (29.5)
BMI (kg/cm^a^cm)	32.12 (7.3)	33.64 (6.0)
DEMMI at screening	15 (3)	15 (3)
TUG at screening^a^	14.88 (9.0)	14.84 (6.1)
Prehab sessions	6 (3)	–
Discharge location^a^: home – rehabilitation center	12 (60) – 6 (30)	10 (50) – 10 (50)
Surgery postponed	2 (10)	

Data are displayed as median (IQR) or frequencies (%). n = number; m = meter; kg = kilogram; BMI = body mass index; cm = centimeter; DEMMI = De Morton Mobility Index; TUG = timed up-and-go test.

a Variable contains missing data.

### 3.3 Preoperative impact

The majority of patients in the intervention group improved between the first and last sessions in terms of their mean scores on all tests (Table 4). The Wilcoxon Signed Rank test showed significant differences between pre- and post-prehabilitation measurements for all secondary outcomes P =<0.05).

**Table 4:** Results regarding preoperative impact.

Test			Wilcoxon Signed Rank test				
	First session	Last session	Z-score	*P*	Last-first session		
					Not improved (n)	Improved (n)	Unchanged (n)
TUG (sec)	19.52 (14.92)	15.85∗	−2.156	0.031	4	15	1
30CST (n)	7.3 (3.3)	10.1∗	−3.035	0.002	2	16	2
2MST (steps)	40.35 (18.1)	52.95∗	−2.461	0.014	5	14	1
2MWT (m)	84.29∗	98.06∗	−2.699	0.007	4	15	1

Data are displayed as means (SD) or ∗ pooled mean in case of imputed data; P = probability; n = number; TUG = timed up-and-go test; sec = seconds; 30CST = 30 s chair stand test; n = repetitions; 2MST = 2 min step test; 2MWT = 2 min walking test; m = meter.

### 3.4 Postoperative impact

The median number of days needed to achieve a modified Iowa level of Assistance scale score of 0 was 4 (3) for the control group, with a range of 3–8 days. The corresponding number for the intervention group was 2 (1); range 1–8 days. The Mann-Whitney *U* Test showed a significantly lower mean rank for the intervention (16.11) group compared to the control group (24.89), with a U score of 14.0 (p=<0.01).

## 4 Discussion

This pilot study investigated the feasibility of conducting a prehabilitation program for 4–6 weeks before surgery in high-risk patients awaiting TKA. We also wanted to explore the impact of this program on the change in preoperative physical fitness and the change in the number of days needed to achieve independence of physical function postoperatively.

The program had a high recruitment rate of 71 % and an adherence rate of 88 %, with a low dropout rate of 12 %; no adverse events were found. Therefore, the prehabilitation program seems safe and feasible. Three patients were forced to stop because of the COVID-19 outbreak. They were excluded from the feasibility analysis because their stopping was unrelated to the content of the program. Prehabilitation focuses on improving preoperative fitness, in order to reduce both time to functional recovery and length of hospitalization. Since these goals are irrelevant to patients with a predetermined discharge date, these patients were also excluded.

The high adherence rate is in line with what was found in the randomized controlled trial by Jetten et al..^37^ Despite their examining a different study population, namely patients awaiting orthotopic liver transplantation, these patients also experienced limitations in physical activity. The authors describe that the high adherence rate was mainly caused by supervision of the sessions. We agree with their conclusion. Supervision during training provides a major incentive.

Preoperatively, the majority of the high-risk patients improved (75 %) on all secondary functional outcomes during the preoperative therapy sessions. This can be seen as a positive impact of the prehabilitation program. This is in agreement with other studies of prehabilitation in patients awaiting total knee arthroplasty.^38^, ^39^, ^40^, ^41^, ^42^, ^43^ Although these studies concerned a general population, not specific high-risk patients as in our sample. Our outcomes show that high-risk patients appear to be trainable too and capable of improvement by participating in a prehabilitation program.

To investigate the postoperative impact of the program, the current intervention group was matched with a comparable retrospective control group, based on DEMMI and TUG scores. Postoperatively, the intervention group achieved in-hospital independence of physical function significantly faster than the control group. One possible explanation could be that the prehabilitation program led to pre-surgical optimization and a better postoperative outcome. This is in agreement with the results of the short-term post-operative effects of prehabilitation for patients awaiting TKA found in the systematic review and meta-analysis by Gränicher et al..^8^ However, another explanation could be that the change in the care pathway, due to continuously changing health care was multi factorial. All the staff involved were more focused on rapid recovery. Resulting in reaching independence of physical function faster. In addition, a possible difference may also have arisen because of the time between groups, a maturation effect.

### 4.1 Strengths

This study had several strengths. First, all data was gathered during usual care. This means that it accurately reflects the care given at that time. No research setting was created, making it the best reflection of the actual situation. The findings of this pilot study are therefore both clinically significant and relevant. Secondly, the intervention and matched control group were perfectly comparable, as can be seen from the median scores on the De Morton Mobility Index and the timed-up-and-go test score, which did not differ significantly between the two groups. Thirdly, the dropout rate was low, which adds power to our findings. The study by Bell et al.^44^ describes that a low dropout rate threatens the validity of results. Dropouts may differ from completers of the program. If there is a low dropout rate we can state with more certainty that the program is feasible and there is a preliminary impact of prehabilitation both preoperatively and postoperatively. Fourthly, the use of a matched control group gave us the opportunity to compare results and provided more insight into the impact of the prehabilitation program. Nevertheless, using a historical cohort can also be seen as a limitation, since a parallel group design was not possible in usual care.

### 4.2 Limitations

The first limitation of this study was that we used a retrospective cohort group, since improvements and optimizations of care could be expected over time. This could cause an overestimation of the effect. For instance, better logistics in 2019–2020 could have led us to overestimate the effect of the prehabilitation program. However, as explained above, since a major change in the care pathway had taken place in 2016 and logistics and care structure have remained the same, we do not expect much change in the time frame we used. Differences between groups over time can also be a result from a maturation effect.^45^ Patients are becoming more experienced with the passage of years, unrelated to the intervention. For example, patients are becoming increasingly informed about the importance of being physically active, which results in a faster discharge to home. These are possible factors influencing the faster achievement of independence of physical function.

Another limitation was the wide variation in the number of sessions attended by each participant. In an optimal situation, patients would have had the opportunity to train for a maximum of 8–10 program sessions, over a period of 4–6 weeks. However, the number of sessions attended by our prehab group varied between 1 and 9, with a median number of 6 (3) sessions. The duration of the program ranged from 3 to 5 weeks. This was due to a shorter interval between selection and surgery, and could have led to underestimation of the impact.

The results of this pilot are generalizable to and implementable in routine clinical care. Our findings show that patients at high risk for prolonged hospital stay are trainable preoperatively, since they can improve their muscle strength and fitness by following a well-designed training program of sufficient duration before surgery. The time available for the training program depends on the waiting list for the surgery in each hospital, but should ideally be between 4 and 6 weeks.^8^ Postoperative impact was also demonstrated, although this will vary with hospital discharge criteria and the definition of the length of stay.

### 4.3 Clinical relevance

We have shown in this pilot that conducting prehabilitation is feasible in a high-risk patient group. We also found that it has an impact on the presurgical physical status and the time to recovery after surgery. Prehabilitation is currently highly relevant in health care, and with the ever-increasing incidence of patients with end-stage KOA, patients will have to be better prepared in order to recover faster and reduce their length of stay.

## 5 Conclusion

Conducting a prehabilitation program for 4–6 weeks before TKA in high-risk patients seems feasible. Participating patients were able to improve their physical fitness during the prehabilitation program, resulting in a positive impact on the presurgical physical status and a faster recovery after surgery. Future research should focus on studies with a parallel group design, large sample sizes, including high-risk patients.

## Funding support

This pilot study did not receive any specific grant from funding agencies in the public, commercial or not-for-profit sectors.

## Declaration of conflicting interests

There are no conflicts of interest.

## Ethical statement

Procedures of data collection were approved by the Ethical Review Board of Maastricht University Medical Center (MUMC+).

## Patient consent

In view of the retrospective study design, in which data were collected during usual care, informed consent was not applicable.

## CRediT authorship contribution statement

**Louisa T.M.A. Mulder:** Conceptualization, Methodology, Investigation, Formal analysis, Writing – original draft, Visualization. **Danielle D.P. Berghmans:** Conceptualization, Supervision, Writing – review & editing. **Peter Z. Feczko:** Supervision, Writing – review & editing. **Rob A. de Bie:** Conceptualization, Supervision, Writing – review & editing. **Antoine F. Lenssen:** Conceptualization, Supervision, Writing – review & editing.
